# Vitamin D binding protein greatly improves bioactivity but is not essential for orally administered vitamin D

**DOI:** 10.14814/phy2.15138

**Published:** 2021-12-07

**Authors:** Elizabeth G. Duchow, Mark W. Duchow, Lori A. Plum, Hector F. DeLuca

**Affiliations:** ^1^ Department of Biochemistry University of Wisconsin‐Madison Madison Wisconsin USA

**Keywords:** ultraviolet light, vitamin D, vitamin D binding protein

## Abstract

Vitamin D_3_ is a prohormone that is essential for calcium homeostasis. It is naturally produced in the skin by ultraviolet‐B (UVB) irradiation of 7‐dehydrocholesterol. In the absence of skin production, vitamin D_3_ can also be obtained from oral sources. However, the actual biological equivalence of naturally produced (i.e., UVB‐irradiation of skin) and oral vitamin D_3_ has not been determined. We previously identified a unique and specific transport mechanism for skin‐generated vitamin D_3_ which requires vitamin D binding protein (DBP); a mechanism that differs from absorption and transport of oral vitamin D_3_. In the following report, we examined the impact of this difference on the biological activity of vitamin D_3_. We report that UVB‐generated vitamin D_3_ is more potent at raising serum calcium compared to oral vitamin D_3_, with the total biological activity being twofold higher. By examining the excretion of radiolabeled vitamin D_3_ injected unbound or pre‐bound by DBP, we attributed the increased activity of skin‐generated vitamin D_3_ to a significant reduction in biliary excretion of DBP‐bound vitamin D relative to unbound vitamin D. Thus, removal of vitamin D_3_ from the skin by the natural DBP system markedly improves biological activity compared to that given orally.

## Graphical abstract

We report that ultraviolet‐B‐generated vitamin D3 is more potent at raising serum calcium compared to oral vitamin D3, with the total biological activity being twofold higher. By examining the excretion of radiolabeled vitamin D3 injected unbound or pre‐bound by D binding protein (DBP), we attributed the increased activity of skin‐generated vitamin D3 to a significant reduction in biliary excretion of DBP‐bound vitamin D relative to unbound vitamin D. Thus, removal of vitamin D3 from the skin by the natural DBP system markedly improves biological activity compared to that given orally.

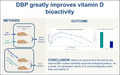

## 1 INTRODUCTION

Vitamin D_3_ is a prohormone that is required for calcium and phosphorous homeostasis. It is naturally produced in the skin from 7‐dehydrocholesterol by exposure to ultraviolet‐B (UVB; 290–315 nm) light (Esvelt et al., 1978). As a secondary source, vitamin D_3_ can also be obtained orally from fish oil, supplements, or fortified foods. Activation of vitamin D_3_ requires enzymatic hydroxylation at the 25‐ and 1‐carbon positions to produce the active hormone, 1*α*,25‐dihydroxyvitamin D_3_ (1,25‐(OH)_2_D_3_). These hydroxylation steps take place in the liver and kidney, respectively (Fraser & Kodicek, 1970; Ponchon et al., 1969). Once activated, 1,25‐(OH)_2_D_3_ stimulates intestinal absorption of calcium and phosphorous enabling normal skeletal mineralization. Furthermore, together with parathyroid hormone, 1,25‐(OH)_2_D_3_ induces calcium mobilization from bone to prevent lethal hypocalcemia.

Although it has traditionally been assumed that oral and cutaneous‐derived vitamin D_3_ are biologically equivalent, they have never been directly compared. Furthermore, work by our laboratory and others have described distinct differences in their transport and delivery to metabolic tissues. Specifically, oral vitamin D_3_ is absorbed from the small intestine and transported to the liver in chylomicrons, whereas we have shown that skin‐generated vitamin D_3_ is transported from skin by vitamin D binding protein (DBP; Avioli, 1969; Duchow et al., 2019; Dueland et al., 1991; Schachter et al., 1964). Upon entering general circulation, oral vitamin D_3_ is rapidly taken up into the liver with a half‐life of ~4–6 h (Mawer et al., 1971). In contrast, blood vitamin D_3_ increases gradually following UVB exposure, peaking at approximately 24 h following exposure and decreases thereafter with a half‐life ranging from 36 to 72 h (Haddad et al., 1993; Stamp et al., 1977).

In the current study, we examined the impact of these differences on the biological activity of vitamin D_3_. We report that the biological activity is greater from skin‐generated vitamin D_3_ than that given orally. By examining the excretion of radiolabeled vitamin D_3_ injected unbound or pre‐bound by DBP, we determined that DBP significantly reduces the amount of vitamin D_3_ excreted in the bile. We also demonstrate that DBP improves the biological activity of oral vitamin D_3_ by comparing the activity in DBP knockout (DBP^−/−^) and wild‐type (DBP^+/+^) mice. Thus, DBP must be considered a major component of the vitamin D system and is essential for naturally produced vitamin D in skin.

## 2 METHODS

### 2.1 Experimental mice

All experiments were conducted in accordance with the Research Animal Resources Committee of the College of Agricultural & Life Sciences, University of Wisconsin–Madison. Animals were maintained in the Department of Biochemistry vivarium with a 12 h:12 h light: dark cycle. Fluorescent bulbs in animal housing and procedure rooms were covered by filters, which eliminate the wavelengths that result in vitamin D_3_ production in skin. DBP^+/−^ mouse embryos were provided by the Cooke laboratory (Perelman School of Medicine, University of Pennsylvania) and rederived at the University of Wisconsin Genomic Editing and Animal Models Core (University of Wisconsin–Madison; Safadi et al., 1999). DBP^+/−^ breeders were maintained on standard laboratory chow 5051 (Purina Mills). Genotyping of offspring was performed by Transnetyx. To generate vitamin D‐deficient animals, DBP^+/+^ and DBP^−/−^ mice were placed on purified diets devoid of vitamin D at the time of weaning and were fed purified diets containing either 0.47% Ca/0.3% P or 0.02% Ca/0.3% P during depletion. The mice were maintained for 1 week on the 0.47% Ca/0.3% P diet, followed by 3 weeks on the 0.02% Ca/0.3% P diet. This was repeated until mice were determined to be deficient by serum calcium and serum 25(OH)D_3_ measurements.

### 2.2 UVB radiation

UVB radiation was carried out as previously described (Irving et al., 2017). The dorsal surface of each mouse was shaved using an electric razor ~24 h before each experiment. Irradiation was performed using a bank of 4 UVB lamps that emit from 280 to 330 nm with a peak at 310 nm (Solarc Systems). The radiation output was measured by placing a UV radiometer equipped with a UVX‐31 sensor with a calibration point of 310 nm and bandpass 280–340 nm (UVP LLC) at 3 locations within the cage to reproduce the positions of the animals. The average output was calculated, and the time was adjusted to ensure exposure to 2.6–8 kJ/m^2^ per treatment.

### 2.3 Skin vitamin D measurement

DBP^−/−^ mice underwent single UVB treatment at the desired dose levels. Mice were euthanized 24 h after treatment by CO_2_ asphyxiation and skin was collected from the entire dorsal surface exposed to UVB light including shaved dorsal skin, ears, and tail. No vitamin D_3_ was detectable in ventral skin. The tissue was minced using razor blades and homogenized in phosphate‐buffered saline (PBS) using a Beadmill24 homogenizer (Fisher Scientific). The entire volume was subjected to a Bligh‐Dyer extraction using CH_2_Cl_2_ in place of CHCl_3_. The CH_2_Cl_2_ layer was dried under argon gas. The remaining oil was dissolved in 2 ml of 5% KOH in 95%methanol/5% H_2_O and saponified for 2 h at 70°C. The lipid soluble saponification products were isolated following the addition of equal volumes of water and hexane. Samples were centrifuged at 3000× g for 5 min. The hexane layer was transferred to a fresh tube, and the sample was washed twice more with equal volumes of hexane. The hexane layers were combined and dried under argon gas. The residual oil was dissolved in 99% hexane/1% isopropanol. 1,2‐[^3^H]‐vitamin D was added prior to each extraction to monitor extraction efficiency. Lipid extracts were applied to a straight‐phase HPLC column (Zorbax SIL, 4.6 × 250 mm, Agilent) run at a flow rate of 0.75 ml/min of 99% hexane/1% isopropanol and monitored at 265 nm. The retention time was based on comigration with the tritiated vitamin D_3_. The limit of detection for this method was 1 ng and the limit of quantification was 2.5 ng.

### 2.4 Serum calcium analysis

Blood was collected from the retro‐orbital sinus for all reported measurements. Blood was collected for baseline serum calcium measurement 24–48 h prior to each experiment and at all indicated timepoints. Serum calcium was determined by atomic absorption using a PerkinElmer 900H spectrophotometer following 1:40 dilution of the serum with 0.1% LaCl_3_. The biological activity was calculated as the area under the curve of the serum calcium plotted over time using GraphPad Prism software.

### 2.5 Dosing solutions and administration

Vitamin D_3_ was purchased from Sigma. Oral vitamin D_3_ was administered in 1% ethanol in Neobee oil via oral gavage. Intravenous injections of free and bound vitamin D were administered in sterile PBS containing 1% ethanol, 0.01% Tween‐20. Recombinant DBP was synthesized by Lytic Solutions, LLC. Briefly, the protein bearing a cleavage his‐tag was expressed by transient transfection of CHO cells and affinity purified by immobilized metal affinity chromatography (IMAC). The purified protein was buffer exchanged and treated with tobacco etch virus (TEV) protease (New England Biolabs) to remove the His‐tag. The cleaved tag, uncleaved protein, and TEV protease (his‐tagged) were removed by passing the digest over an IMAC column.

Radiolabeled vitamin D_3_ was synthesized by Moravek Inc., To generated [3H]‐vitamin D‐DBP complexes, binding was carried out at the desired dosing solution concentration with 6 µg of DBP in 0.01% Tween‐20, 1% ethanol in sterile PBS overnight at 4°C. Unbound [3H]‐vitamin D was removed on a Sephadex G‐25 spin column and flow‐through was used as the dosing solution. For the “free vitamin D” dosing solution, [3H]‐vitamin D was incubated in 0.01% Tween‐20, 1% ethanol in sterile PBS overnight at 4°C. The dosing solution concentrations were confirmed by liquid scintillation counting using a TRI‐CARB 4810 TR liquid scintillation analyzer (PerkinElmer). Intravenous injections were administered via retro‐orbital injection under ether anesthesia. Oral doses were administered via oral gavage.

### 2.6 Fecal analysis for biliary excretion

Mice were housed in metabolic cages for feces and urine collection for 24–48 h. Dried feces were collected, rehydrated in 0.5 ml of H_2_O, and solubilized in 2 ml tissue solubilizer for 2 h at 50°C. Samples were decolorized with 0.2 ml 30% H_2_O_2_. The tritium content of solubilized samples was determined by liquid scintillation counting using a TRI‐CARB 4810 TR liquid scintillation analyzer (PerkinElmer).

### 2.7 Statistical analysis

Data are expressed as mean ± *SEM*. Statistical analysis was performed using the mixed model procedure with Tukey's adjustment using SAS Version 9.4 (SAS Institute). A value of *p* < 0.05 was considered statistically significant.

## 3 RESULTS

### 3.1 UVB‐generated vitamin D_3_ is more biologically active than orally administered vitamin D_3_

The biological activity of an equivalent amount of vitamin D_3_ generated either from a single UVB exposure or delivered as a single oral dose was compared in vitamin D‐deficient, hypocalcemic mice. The biological activity was assessed based on the area under the curve of the serum calcium response over time for individual animals and averaged within each group.

The biological activity was evaluated for “low” and “high” dosages. To determine the required oral dose, the total amount of vitamin D_3_ produced by the UVB conditions was measured in vitamin D‐deficient DBP^−/−^ mice since the vitamin D_3_ does not leave the skin once generated in these mice (Duchow et al., 2019). To obtain this measurement, lipids were isolated from skin collected from the entire dorsal surface exposed to UVB light including dorsal skin, ears, and tail skin. No vitamin D_3_ was detectable in ventral skin. Vitamin D_3_ content was determined to be 10 ng/mouse and 34 ng/mouse at the low and high UVB dose levels, respectively (*n* = 5 for each dose level).

**FIGURE 1:**
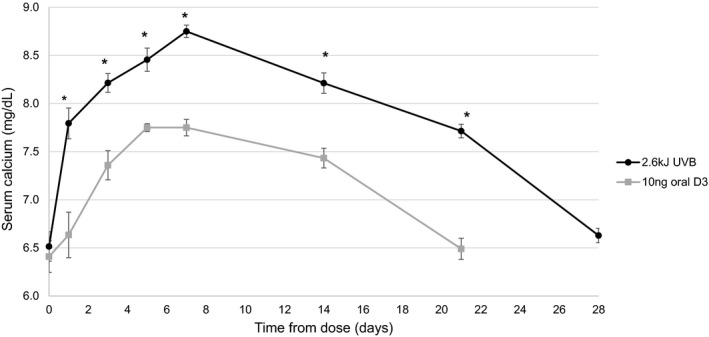
Serum calcium response following a single UV treatment or a single oral dose with an equivalent amount of vitamin D_3_. UV‐generated vitamin D_3_ resulted in an increased and more sustained response compared to a single oral dose of vitamin D_3_. Vitamin D‐deficient hypocalcemic mice underwent a single treatment with 2.6 kJ ultraviolet‐B (UVB; previously determined to produce 10 ng of vitamin D_3_) or received a single oral dose of 10 ng vitamin D_3_ in Neobee oil via oral gavage. Blood was collected for baseline calcium measurement 48 h prior to treatment (*n* = 4/group/timepoint for days 1–7, *n* = 8/group/timepoint for day 0 and days 8–28, ±*SEM*, *p < 0.05 vs. 10 ng oral D_3_)

At the low dose, treatment with both UVB (2.6 kJ) and oral vitamin D_3_ (10 ng) resulted in an increase in serum calcium, with UVB‐generated vitamin D_3_ being significantly more potent at raising serum calcium in wild‐type mice (Figure 1). The area under the curve for serum calcium response over time was approximately twofold higher for UVB‐generated vitamin D_3_ compared to oral vitamin D_3_ (40 ± 4 mg × d/dl vs. 17 ± 2 mg × d/dl). The initial response to the treatment was more rapid for UVB‐generated vitamin D_3_, occurring within 24 h of dosing, whereas an increase was not detectable until 72 h after oral administration. Vitamin D_3_ generated by the low UVB dose was sufficient to normalize serum calcium levels (8.8 ± 0.1 mg/dl). However, when administered orally, the maximum level achieved was 7.8 ± 0.1 mg/dl. Serum calcium gradually returned to baseline level by day 21 for the oral treatment group and day 28 for the UVB group.

**FIGURE 2:**
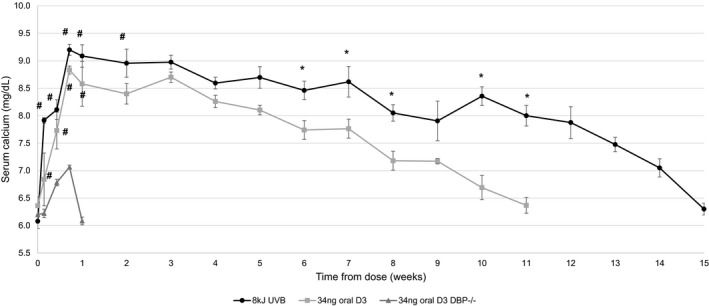
Serum calcium response following a single UV treatment or a single oral dose with an equivalent amount of vitamin D_3_. Serum calcium response to a single UV treatment was greater and more sustained compared to a single oral dose of vitamin D_3_ in DBP^+/+^ and DBP^−/−^ mice. Vitamin D‐deficient hypocalcemic mice underwent a single treatment with 8 kJ UVB (previously determined to produce 34 ng of vitamin D_3_) or received a single oral dose of 34 ng vitamin D_3_ in Neobee oil via oral gavage. Blood was collected for baseline calcium measurement 48 h prior to treatment (*n* = 6/group, ±*SEM*, *p < 0.05 vs. 34 ng oral D_3_, ^#^ *p* < 0.05 vs. 34 ng oral D_3_ DBP^−/−^). DBP, D binding protein; UVB, ultraviolet‐B

Similar results were obtained at the high UVB (8 kJ) and oral (34 ng) dose (Figure 2). The area under the curve was 254 ± 30 mg × d/dl for the UVB group and 127 ± 19 mg × d/dL for the oral dose group. As found with the low dose experiment, a significant increase occurred within 24 h after UVB treatment, whereas an increase was not detectable until the 72‐h timepoint for the wild‐type oral dose group. After the 24‐h timepoint, serum calcium remained comparable between the two groups (difference <0.6 mg%) through week 5. Serum calcium for the oral treatment group began to gradually decline after week 5 and returned to baseline at 12 weeks in DBP^+/+^ mice. In contrast, serum calcium levels remained elevated in the UVB group through week 7 and returned to baseline at week 15.

The impact of DBP on the biological activity of oral vitamin D_3_ was also assessed in vitamin D‐deficient and hypocalcemic DBP^−/−^ mice at the high oral dose (Figure 2). As expected, activity was significantly reduced in DBP^−/−^ mice compared to DBP^+/+^ mice. The area under the biological activity curve was approximately 2.8 ± 0.6 mg × d/dl, an amount approximately 40 times lower than DBP^+/+^ mice. Besides the much lower maximal response (7.1 mg/dl vs. 8.8 mg/dl), oral administration in DBP^−/−^ mice also resulted in a very quick return to baseline (1 week vs. 11 weeks).

### 3.2 DBP improves the biological activity of oral vitamin D3

**FIGURE 3:**
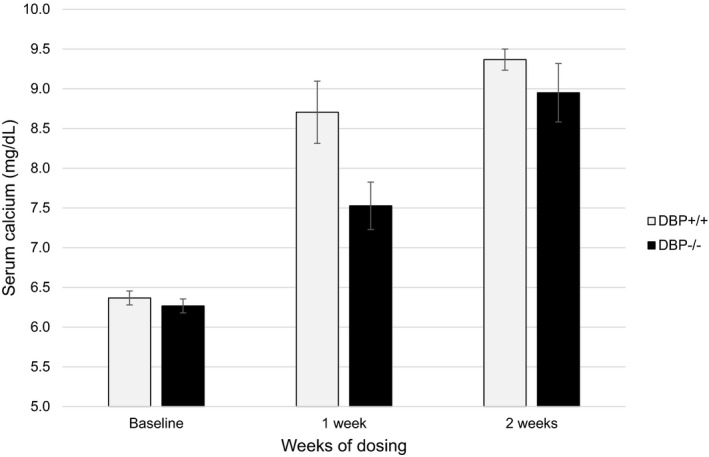
Serum calcium levels after daily oral administration of vitamin D_3_ in vitamin D deficient and hypocalcemic DBP^+/+^ and DBP^−/−^ mice. DBP^−/−^ mice required an additional week of oral dosing to normalize serum calcium levels relative to DBP^+/+^ mice. Vitamin D‐deficient and hypocalcemic mice received daily oral doses of 0.25 µg vitamin D_3_. Blood was collected for baseline calcium measurement 24 h before initial treatment (*n* = 3/group, ±*SEM*). DBP, D binding protein

To further investigate the decreased activity of oral vitamin D_3_ in DBP^−/−^ mice relative to DBP^+/+^ mice, the serum calcium response to daily oral administration of a physiologic dose of vitamin D_3_ (250 ng) was compared in vitamin D‐deficient mice. After 1 week of dosing, serum calcium levels had normalized in DBP^+/+^ mice (6.4 ± 0.1 mg/dl vs. 8.7 ± 0.4 mg/dl) and had increased from 6.3 ± 0.1 mg/dl to 7.5 ± 0.3 mg/dl in DBP^−/−^ mice (Figure 3). DBP^−/−^ mice required 2 weeks of daily dosing to normalize serum calcium (9.0 ± 0.4 mg/dl). At the end of the treatment, serum calcium levels were 9.4 ± 0.1 and 9.0 ± 0.4 mg/dl for the DBP^+/+^ and DBP^−/−^ mice, respectively.

### 3.3 DBP mitigates excessive biliary excretion of [3H]‐vitamin D3

**FIGURE 4:**
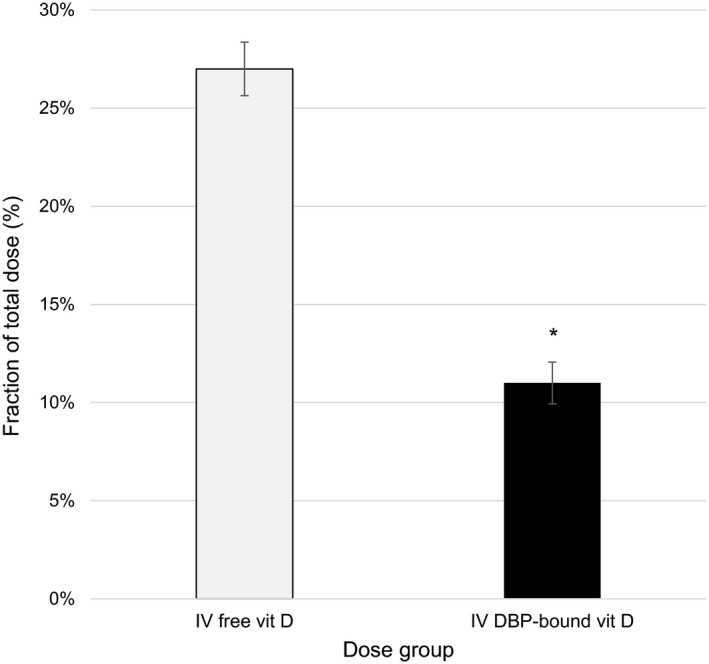
Fraction of total dose of radiolabeled vitamin D_3_ present in the feces of mice 24‐h after injection in DBP^+/+^ mice. Injection of vitamin D_3_ pre‐bound by DBP resulted in a ~2.5‐fold decrease in total radiolabel excreted over 24 h. Vitamin D sufficient mice received single IV injection of 8 ng [3H]‐vitamin D either unbound or pre‐bound to DBP (*n* = 3/group, ±*SEM*, *p < 0.05 vs. IV free vit D). DBP, D binding protein

To determine if the differences in the potency and duration of activity were due to changes in clearance by the liver, we evaluated the biliary excretion of tritium‐labeled vitamin D_3_ in vitamin D sufficient wild‐type mice. Intravenous injection of 8 ng of [^3^H]‐vitamin D_3_ pre‐bound to DBP resulted in a 2.5‐fold reduction in the total amount of tritium detectable in the feces at 24 h relative to injection of 8 ng of unbound [^3^H]‐vitamin D_3_ (Figure 4). The total fraction of the dose excreted in the feces was 27% in mice that received free vitamin D_3_, whereas this was only 11% in mice injected with vitamin D_3_ pre‐bound by DBP.

**FIGURE 5:**
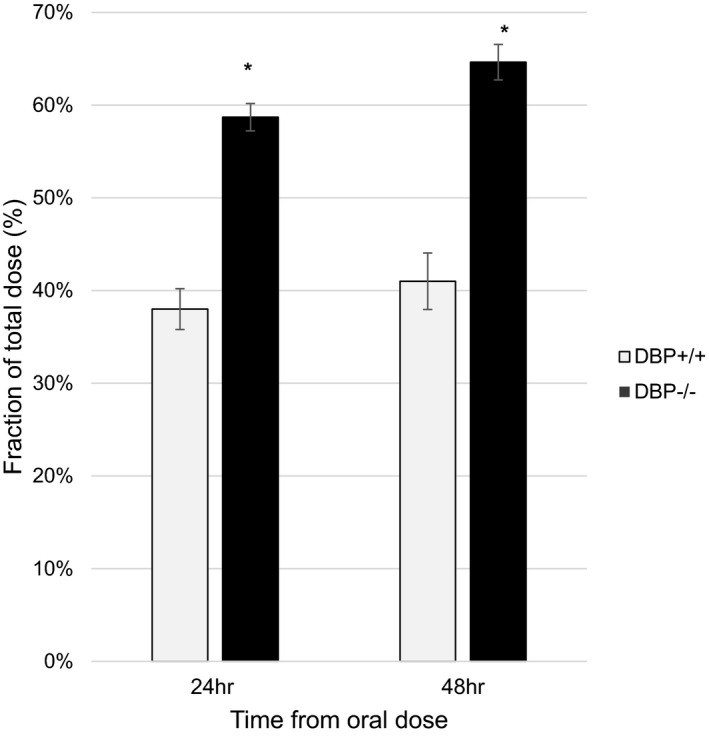
Fraction of total dose of radiolabeled vitamin D present feces of DBP^+/+^ and DBP^−/−^ mice after dosing. Excretion of oral [3H]‐vitamin D is significantly higher in DBP^−/−^ mice at 24 and 48‐h following administration. Vitamin D sufficient mice received a single oral dose of 0.25 µg [3H]‐vitamin D (*n* = 3/group, ±*SEM*, *p < 0.05 vs. DBP^+/+^). DBP, D binding protein

We also examined excretion of a single oral dose of radiolabeled vitamin D_3_ by DBP^−/−^ mice compared to DBP^+/+^ mice. After 24 h, a larger portion of the dose was detectable in the feces of DBP^−/−^ mice compared to the DBP^+/+^ mice (Figure 5, 38% vs. 59%). By 48 h, this had increased to 41% of the dose in DBP^+/+^ mice and 65% in DBP^−/−^ mice.

## 4 DISCUSSION

Our results demonstrate that DBP significantly increases the biological activity of vitamin D_3_. Although orally administered vitamin D_3_ is biologically effective, its potency and the duration of activity are reduced compared to that delivered to the body bound to DBP. This difference is at least in part due to the decreased biliary excretion of DBP‐bound vitamin D_3_. As a result, the activity of oral vitamin D_3_ is significantly reduced compared to UVB‐generated vitamin D_3_ which we previously showed is delivered exclusively by DBP (Duchow et al., 2019). Additional evidence of the importance of DBP for optimal vitamin D_3_ activity is exemplified in DBP^−/−^ mice where the potency of oral vitamin D_3_ is dramatically reduced, likely because of rapid clearance of unbound vitamin D_3_ from the body.

The increased biliary excretion of vitamin D_3_ observed with free and oral vitamin D_3_ is likely a result of the rapid accumulation in the liver leading to its degradation. This is consistent with a previous report that excessive accumulation of vitamin D_3_ in the liver results in the appearance of water‐soluble metabolites (Fraser, 1983). Investigation of hepatic uptake through liver perfusion of radiolabeled vitamin D_3_ bound by its various carriers in rats indicated that uptake is significantly reduced when bound by DBP. Therefore DBP transport may keep vitamin D_3_ out of the liver, preventing accumulation and protecting it from rapid degradation (Haddad et al., 1988).

We also found that the duration of biological activity is significantly longer with UVB‐generated vitamin D_3_ relative to oral vitamin D_3_, suggesting a difference in the metabolism. Previous investigations of vitamin D_3_ metabolism after oral administration report a spike in 25‐OHD_3_ levels shortly after administration (Mawer et al., 1971; Stamp et al., 1977). In contrast, a gradual and sustained increase in 25‐OHD_3_ levels has been reported following UVB treatment (Adams et al., 1982; Mawer et al., 1971; Stamp et al., 1977). The prolonged production of 25‐OHD_3_ likely leads to continued generation of biologically active 1,25‐(OH)_2_D_3_, thus increasing the bioactivity as seen in these experiments.

In addition to sustained biological activity, the observed potency was higher for UVB‐generated vitamin D_3_ than oral vitamin D_3_. At the low dose, oral vitamin D did not restore normal blood calcium levels, whereas UV administration did. An oral dose of vitamin D three times higher was necessary to achieve normal circulating levels of calcium. We propose the increased potency is due to a larger pool of bioavailable vitamin D_3_ resulting in the production of larger amounts of 1,25‐(OH)_2_D_3_ when transported by DBP. However, as 25‐OHD_3_ and 1,25‐(OH)_2_D_3_ were not measured in these experiments, this cannot be definitively determined.

The observation that physiologic doses of vitamin D_3_ are approximately half as effective at correcting vitamin D_3_ deficiency in DBP^−/−^ mice support the concept that DBP is important for the biological activity of oral vitamin D_3_ albeit not essential. The increased biliary excretion observed in DBP^−/−^ mice relative to DBP^+/+^ is interesting because DBP is not involved in absorption of vitamin D_3_. Therefore, the rate of delivery to the liver should be comparable between the two genotypes. Considering this, it is possible that DBP may be required for optimal removal of 25‐OHD_3_ following its production in the liver, preventing its degradation. It is also possible that DBP binding in the liver sequesters 25‐OHD_3_, preventing further metabolism.

These data demonstrate that oral vitamin D_3_ and UVB‐generated vitamin D_3_ are not biologically equivalent, and that vitamin D_3_ derived through the natural (skin) system is more biologically active. Our results are suggestive that the non‐specific absorption of oral vitamin D_3_ and transport to the liver results in excessive waste as biliary excretion, whereas DBP‐bound vitamin D_3_ is protected.

## CONFLICT OF INTEREST

The authors have nothing to disclose.

## AUTHOR CONTRIBUTIONS

E.G.D., L.A.P., and H.F.D designed the experiments. E.G.D. performed research. M.W.D. maintained experimental animals. E.G.D, L.A.P., and H.F.D. analyzed the data. E.G.D., L.A.P., and H.F.D wrote the paper.

## References

[phy215138-bib-0001] Adams, J. S. , Clemens, T. L. , Parrish, J. A. , & Holick, M. F. (1982). Vitamin‐D synthesis and metabolism after ultraviolet irradiation of normal and vitamin‐D‐deficient subjects. New England Journal of Medicine, 306, 722–725. 10.1056/NEJM198203253061206 7038486

[phy215138-bib-0002] Avioli, L. V. (1969). Absorption and metabolism of vitamin D_3_ in man. American Journal of Clinical Nutrition, 22, 437–446. 10.1093/ajcn/22.4.437 4305087

[phy215138-bib-0003] Duchow, E. G. , Cooke, N. E. , Seeman, J. , Plum, L. A. , & DeLuca, H. F. (2019). Vitamin D binding protein is required to utilize skin‐generated vitamin D. Proceedings of the National Academy of Sciences of the United States of America, 116, 24527–24532. 10.1073/pnas.1915442116 31748273 PMC6900711

[phy215138-bib-0004] Dueland, S. , Nenseter, M. S. , & Drevon, C. A. (1991). Uptake and degradation of filamentous actin and vitamin‐D‐binding protein in the rat. Biochemical Journal, 274, 237–241. 10.1042/bj2740237 PMC1149943 2001239

[phy215138-bib-0005] Esvelt, R. P. , Schnoes, H. K. , & DeLuca, H. F. (1978). Vitamin D_3_ from rat skins irradiated in vitro with ultraviolet light. Archives of Biochemistry and Biophysics, 188, 282–286. 10.1016/S0003-9861(78)80010-1 209750

[phy215138-bib-0006] Fraser, D. R. (1983). The physiological economy of vitamin D. Lancet, 1, 969–972. 10.1016/S0140-6736(83)92090-1 6132277

[phy215138-bib-0007] Fraser, D. R. , & Kodicek, E. (1970). Unique biosynthesis by kidney of a biological active vitamin D metabolite. Nature, 228, 764–766. 4319631 10.1038/228764a0

[phy215138-bib-0008] Haddad, J. G. , Jennings, A. S. , & Aw, T. C. (1988). Vitamin D uptake and metabolism by perfused rat liver: Influences of carrier proteins. Endocrinology, 123, 498–504. 2838261 10.1210/endo-123-1-498

[phy215138-bib-0009] Haddad, J. G. , Matsuoka, L. Y. , Hollis, B. W. , Hu, Y. Z. , & Wortsman, J. (1993). Human plasma transport of vitamin D after its endogenous synthesis. Journal of Clinical Investigation, 91, 2552–2555. 10.1172/JCI116492 PMC443317 8390483

[phy215138-bib-0010] Irving, A. A. , Marling, S. J. , Plum, L. A. , & DeLuca, H. F. (2017). Suppression of experimental autoimmune encephalomyelitis by ultraviolet light is not mediated by isomerization of urocanic acid. BMC Neuroscience, 18, 8. 10.1186/s12868-016-0323-2 28056806 PMC5217575

[phy215138-bib-0011] Mawer, E. B. , Lumb, G. A. , Schaefer, K. , & Stanbury, S. W. (1971). Metabolism of isotopically labelled vitamin‐D3 in man—Influence of state of vitamin‐D nutrition. Clinical Science, 40, 39–53. 4321723 10.1042/cs0400039

[phy215138-bib-0012] Ponchon, G. , Kennan, A. L. , & DeLuca, H. F. (1969). "Activation" of vitamin D by the liver. Journal of Clinical Investigation, 48, 2032–2037. 10.1172/JCI106168 PMC297455 4310770

[phy215138-bib-0013] Safadi, F. F. , Thornton, P. , Magiera, H. , Hollis, B. W. , Gentile, M. , Haddad, J. G. , Liebhaber, S. A. , & Cooke, N. E. (1999). Osteopathy and resistance to vitamin D toxicity in mice null for vitamin D binding protein. Journal of Clinical Investigation, 103, 239–251. 10.1172/JCI5244 PMC407885 9916136

[phy215138-bib-0014] Schachter, D. , Finkelstein, J. D. , & Kowarski, S. (1964). Metabolism of vitamin D. I. Preparation of radioactive vitamin D and its intestinal absorption in the rat. Journal of Clinical Investigation, 43, 787–796. 10.1172/JCI104965 PMC289558 14169508

[phy215138-bib-0015] Stamp, T. C. B. , Twigg, C. A. , & Haddad, J. G. (1977). Comparison of oral 25‐hydroxycholecalciferol, vitamin‐D, and UV light as determinants of circulating 25‐hydroxyvitamin‐D. The Lancet, 1, 1341–1343. 10.1016/S0140-6736(77)92553-3 69059

